# Probing the thermal decomposition mechanism of CF_3_SO_2_F by deep learning molecular dynamics

**DOI:** 10.1038/s42004-025-01847-x

**Published:** 2025-12-19

**Authors:** Anyang Wang, Zeyuan Li, Shubo Ren, Xue Ke, Xuhao Wan, Rong Han, Xianglian Yan, Wen Wang, Yu Zheng, Yuzheng Guo, Jun Wang

**Affiliations:** 1 School of Electrical Engineering and Automation, Wuhan University, Wuhan, China; 2 School of Power and Mechanical Engineering, Wuhan University, Wuhan, China; 3 High Voltage Department, China Electric Power Research Institute, Beijing, China

**Keywords:** Molecular dynamics, Atomistic models, Reaction mechanisms

## Abstract

The urgent need to phase out SF_6_, an extremely potent greenhouse gas prevalent in electrical grids, drives the search for eco-friendly insulation alternatives. Trifluoromethanesulfonyl fluoride (CF_3_SO_2_F) emerges as a promising candidate due to its excellent properties. However, understanding its thermal decomposition pathways and products under operationally relevant conditions is critical for evaluating its environmental feasibility and mitigating potential risks upon accidental release or during fault events. This study investigates the thermal decomposition mechanisms of CF_3_SO_2_F using a deep learning potential that combines ab initio accuracy with empirical MD efficiency. By leveraging machine learning driven molecular dynamics, we systematically analyze the yields and components of decomposition products versus temperatures, gas mixing ratios, and buffer gas. The results reveal that the bond-breaking pathways are temperature-dependent, with both elevated temperatures and higher buffer gas mixing ratios promoting its decomposition. Elevated gas pressure enhances the decomposition process by increasing the collision frequency among reactant species. Additionally, N_2_ exhibits an inhibitory effect on decomposition under high pressure compared to CO_2_. Experimental validation via a thermal decomposition platform confirms characteristic decomposition products. These findings are pivotal for guiding the rational design and safe deployment of CF_3_SO_2_F to achieve substantial greenhouse gas mitigation in the power industry.

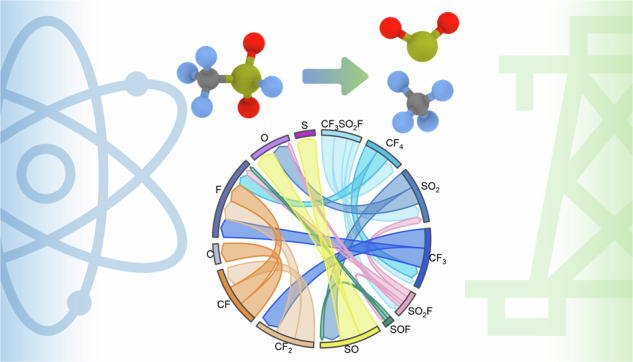

## Abstract (web summary)

Trifluoromethanesulfonyl fluoride (CF_3_SO_2_F) is a promising alternative to SF_6_, a potent greenhouse gas prevalent in electrical grids, but understanding its decomposition pathways and products is crucial for evaluating its environmental feasibility. Here, the authors investigate its thermal decomposition using machine learning-driven molecular dynamics, revealing temperature- and gas pressure-dependent bond-breaking pathways and determining and experimentally validating its decomposition products.

## Introduction

Insulating gases play an irreplaceable role in power systems. Sulfur hexafluoride (SF_6_) has been widely used as an insulating gas in power equipment such as switchgear and transmission pipelines due to its excellent insulation and arc extinguishing properties^1,2^. However, the potential greenhouse effect caused by high global warming potential (GWP = 23900) and atmospheric lifetime (3200 years) of SF_6_ will have an irreversible impact on the atmosphere and the earth’s environment, and it is imperative to explore the green and eco-friendly new insulation medium^3–6^. Researchers have done much work in SF_6_ alternative gases^7,8^. So far, some gases with comparatively high insulating properties, such as CF_3_I, C_5_F_10_O, and C_4_F_7_N, and their mixtures are being investigated as SF_6_ replacement gases^9–11^. However, they have different defects in dielectric strength, liquefaction temperature, GWP, toxicity, stability and so on. Even C_4_F_7_N, the most popular gas in SF_6_ substitution research, faces the challenge of high liquefaction temperature, making its application in alpine areas difficult^12^.

In recent years, trifluoromethylsulfonyl fluoride (CF_3_SO_2_F) has proven to be an eco-friendly insulation replacement gas with excellent potential and performance. Not only the AC and DC breakdown voltage of CF_3_SO_2_F can be increased to 1.3–1.6 times that of SF_6_ under the same conditions, but also the GWP of CF_3_SO_2_F (3678) is much lower than that of SF_6_^13,14^. In addition, the lower liquefaction temperature (–22 °C), excellent gas-solid compatibility, low toxicity, and high stability of CF_3_SO_2_F have been proven^15,16^. However, gaseous dielectrics can decompose under discharge or localized overheating faults, forming inevitable byproducts. The study of the decomposition characteristics of gas-insulating media is an essential indicator of its reliability, closely related to its self-recovery and insulation performance^17–19^. Wang et al. analyzed the possible decomposition pathways and byproducts of CF_3_SO_2_F by quantum chemical methods^15^. Our team calculated the equilibrium composition, thermodynamic properties, and transport coefficients of CF_3_SO_2_F gas mixtures and found that the gas mixing ratio and pressure affect the thermophysical and transport properties of CF_3_SO_2_F gas mixtures^20,21^. Despite these contributions, current kinetic and thermodynamic studies present fundamental limitations. Existing research often overlooks the dynamic decomposition properties of CF_3_SO_2_F, especially under varying conditions, which impedes risk assessment and safe deployment^22^. The experimental difficulties and high costs associated with such studies exacerbate these gaps, resulting in a lack of comprehensive insights into the micro-mechanisms underlying these processes^23,24^.

Theoretical approaches are powerful tools to study the dynamic decomposition properties of CF_3_SO_2_F mixtures at the atomic level^15,25^. The molecular simulation community has long faced the problem of accuracy and efficiency in modeling potential energy surfaces and interatomic forces. For instance, although the ab initio molecular dynamics (AIMD) simulations based on density functional theory (DFT) could show sufficient accuracy in describing chemical reactions that contain bond cleavage and formation, its computational cost of such high-level methods would limits their application to systems with hundreds to thousands of atoms^26,27^. Conversely, empirical and reactive force fields allow for larger and longer simulations^24,28–31^. However, parameters fitting in these methods are usually lengthy processes, and their accuracy and transferability are often questioned. Therefore, a theoretical approach with quantum chemical accuracy and lower computational resource requirements is needed for high-precision simulations of the dynamic thermal decomposition process of CF_3_SO_2_F gas mixtures. Over the past few years, machine learning methods using DFT data have achieved some notable successes in the characterization of molecular macrosystems^32–34^. Deep learning potential (DLP) developed on machine learning can automatically extract features from DFT data for deep neural network training and achieve preset accuracies^35,36^. DLP can take into account the time cost of empirical force fields while maintaining accuracy in ab initio. Yang et al. have successfully used DLP to simulate the dynamic and complex decomposition process of urea in water^37^.

Inspired by machine learning-based approaches to molecular simulations, a neural network-based machine learning potential with comparable accuracy to ab initio and comparable efficiency to empirical potential-based molecular dynamics was developed to describe the dynamic thermal decomposition of CF_3_SO_2_F mixtures. Based on the machine-learning potential-driven molecular dynamics (MD) simulations, the decomposition mechanism of CF_3_SO_2_F mixtures at different temperatures and gas mixing ratios and components of the main decomposition products were obtained. The effects of the CF_3_SO_2_F mixing ratio on the decomposition of the CF_3_SO_2_F mixture were analyzed to reveal the mechanism of the thermal decomposition of the CF_3_SO_2_F mixture under different pressures. Compared with CO_2_, N_2_ as a buffer gas can inhibit the decomposition of CF_3_SO_2_F to a certain extent. The thermal decomposition properties of CF_3_SO_2_F gas mixtures were experimentally investigated using a constructed thermal decomposition platform, and characteristic decomposition products aiming to characterize the failure were proposed. This work provides significant insight into the thermal decomposition behavior of CF_3_SO_2_F and its mixtures, contributing to the understanding of its stability and decomposition pathways under extreme conditions. Furthermore, the computational framework is potentially transferable for investigating the physicochemical properties and decomposition mechanisms of other promising insulating gases, guiding the development of eco-friendly alternatives to SF_6_.

## Results

### DLP training and structure of simulation boxes

**Fig. 1:**
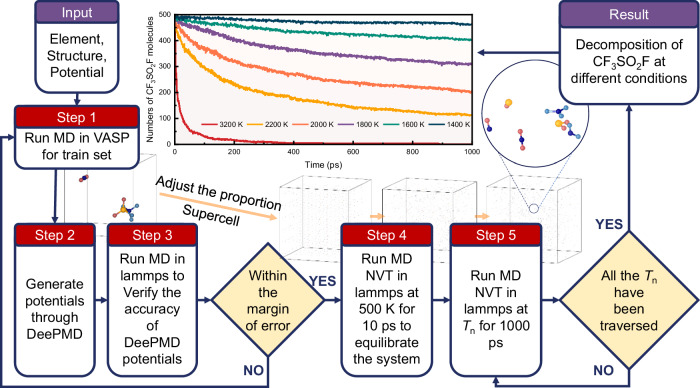
Workflow for constructing and validating the deep learning potential for CF_3_SO_2_F decomposition. The workflow comprises four key steps: (1) Configuration sampling from AIMD trajectories, (2) Training of the DLP, (3) Validation of the DLP against DFT benchmarks, and (4) Large-scale molecular dynamics simulations of CF_3_SO_2_F decomposition across a range of temperatures (T_1_ to T_n_), with supercell size adjusted to model different system pressures.

Figure 1 illustrates the workflow involved in training DLP and calculating the decomposition products of CF_3_SO_2_F at various temperatures based on DLP. Initially, a potential is trained on a dataset composed of structures randomly selected from the AIMD reaction trajectories of a model with well-determined elemental and molecular configurations. This initial potential serves as a starting point for an iterative training process. Subsequently, the DLP of CF_3_SO_2_F is developed and trained using potentials, forces, and virials from the AIMD dataset, which consists of a temperature range from 300 K to 3200 K in the NVT ensemble^35^. Through multiple explorations, labeling, and training, we compiled a comprehensive dataset of the CF_3_SO_2_F gas mixture configurations across a wide range of temperatures^27,38^. The DLP was trained and fine-tuned after the dataset was built, and extensive validation tests confirmed that it would produce only small errors. Detailed information on the settings used for AIMD computation and DLP training is given in the methods section.

The reliability of the deep learning potential for modeling reactive events was rigorously validated. Initially, a machine-learning potential was trained on a dataset constructed by systematically sampling structures at regular time intervals from the AIMD reaction trajectories. This procedure ensures that the dataset captures a representative ensemble of the well-defined, chemically relevant configurations sampled during the reactive dynamics, covering both stable intermediates and transition regions, as shown in Fig. S1. The dataset is randomly divided into a training set (comprising 90% of the structures) and a validation set (comprising 10% of the structures). The training set was employed to fit a neural network, with internal validation performed using the validation set to ensure that the error on the validation set is not significantly higher than that on the training set. As illustrated in Fig. S2, the trained potential function accurately reproduces the DFT data, yielding a root-mean-square error (RMSE) of merely 1.3 meV for the total energy of each atom. The RMSE for the forces is 0.023 eV/Å, and the RMSE for the virials per atom is 2.5 meV, closely aligning with the DFT results. The deviation distribution plots for energy, force, and virials are all centered around zero, indicating the model’s robust predictive capability.

To quantitatively assess the potential’s accuracy, the potential energy surfaces of the CF_3_SO_2_F molecule with changing CF_3_−SO_2_F, F−CF_2_SO_2_F, F−CF_3_SO_2_, and O−CF_3_SOF bond lengths were computed with both the DLP and the reference DFT method, showing excellent agreement (Fig. S3). Moreover, the DLP’s description of key stationary points was consistent with high-level CCSD(T) reference data from the literature^15^. These results consistently identify C–S bond homolysis as the dominant initial decomposition step, followed by dissociation of the resulting radical, which confirms its transferability and robustness for probing the decomposition mechanism. The principal advantage of the DLP approach lies in its capacity to go beyond static energy calculations and provide statistically meaningful insights into finite-temperature reaction dynamics, including product branching ratios, at a computational cost inaccessible to direct ab initio molecular dynamics. Finally, the trained DLP was utilized to simulate the CF_3_SO_2_F/CO_2_ gas mixture (hereafter denoting a mixture of CF_3_SO_2_F in a CO_2_ buffer gas) at various temperatures.

Molecular dynamics simulations using the trained DLP were conducted with LAMMPS^39^. Seven initial simulation boxes under different states, which contain at least 10,000 atoms to simulate the real environment, were created by randomly placing the molecules in a cubic box, as detailed in Table 1. For instance, simulation box No. 3 comprises 500 CF_3_SO_2_F molecules and 3667 CO_2_ molecules, totaling 15,001 atoms, with a cubic box side length of 553.24 Å and a 0.002328 g/cm³ density. This configuration corresponds to an actual condition of a 12% CF_3_SO_2_F/CO_2_ gas mixture at 25 °C and 0.1 MPa.

**Table 1:** Parameters of the simulated CF_3_SO_2_F/CO_2_ systems

No	P	mixing ratio	CF_3_SO_2_F	CO_2_	number of atoms	Density (g/cm3)	box length/Å
1	0.1 MPa	0.20	500	2000	10000	0.002681	466.6134
2	0.1 MPa	0.14	500	3071	13213	0.002417	525.5068
3	0.1 MPa	0.12	500	3667	15001	0.002328	553.2428
4	0.3 MPa	0.12	500	3667	15001	0.006985	383.5971
5	0.5 MPa	0.12	500	3667	15001	0.011642	323.5384
6	0.1 MPa	0.14	583	3584	15416	0.002681	553.2428
7	0.1 MPa	0.20	833	3334	16666	0.002417	553.2428

The pressure listed is the initial ideal gas pressure calculated from the given number of molecules in the simulation cell at 300 K.

It has been demonstrated that CF_3_SO_2_F gas mixtures with less than 20% CF_3_SO_2_F mixing ratio are more advantageous for engineering applications^40,41^. To investigate the decomposition of CF_3_SO_2_F gas mixtures under different mixing ratios and the impact of these mixing ratios on decomposition, simulations were performed with varying CF_3_SO_2_F mixing ratios. No.1 and No.2 simulation boxes maintain the total CF_3_SO_2_F molecules fixed and simulate 20% CF_3_SO_2_F/CO_2_ and 14% CF_3_SO_2_F/CO_2_ systems, respectively. Similarly, No.6 and No.7 simulation boxes keep the total number of molecules constant to simulate the same systems. In addition, simulation boxes No. 4 and No. 5 represent the decomposition of 12% CF_3_SO_2_F/CO_2_ gas mixture at 0.3 MPa and 0.5 MPa to investigate the effect of pressure on decomposition respectively. The simulation boxes for CF_3_SO_2_F/N_2_ (hereafter denoting a mixture of CF_3_SO_2_F in a N_2_ buffer gas) under different conditions were built according to the same approach as shown in Table S1.

### Decomposition of CF_3_SO_2_F

**Fig. 2:**
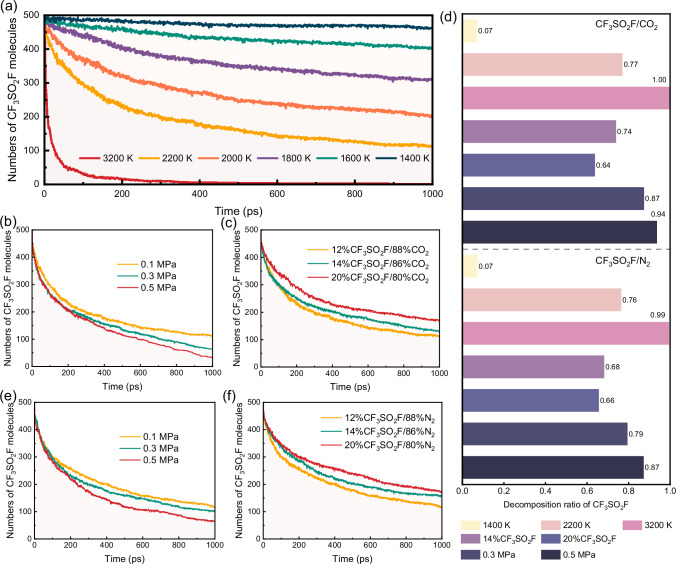
Thermal decomposition of CF_3_SO_2_F under various conditions. **a** Decomposition time evolution at 0.1 MPa with different temperatures in 12%CF_3_SO_2_F/88%CO_2_ mixture. **b** Decomposition time evolution at 2200 K with different pressures in 12%CF_3_SO_2_F/88%CO_2_ mixture. **c** Decomposition time evolution at 2200 K and 0.1 MPa with different mixing ratios in CF_3_SO_2_F/CO_2_ mixtures. **d** Final decomposition ratios after 1000 ps under different conditions in CF_3_SO_2_F/CO_2_ and CF_3_SO_2_F/N_2_ mixtures. **e** Decomposition time evolution at 2200 K with different pressures in 12%CF_3_SO_2_F/88%N_2_ mixture. **f** Decomposition time evolution at 2200 K and 0.1 MPa with different mixing ratios in CF_3_SO_2_F/N_2_ mixtures.

The two primary causes of decomposition in the insulating dielectric region are localized overheating faults and high temperatures resulting from localized (corona) or arc discharges^24^. The temperature in the core region of a localized discharge ranges from 700 to 1200 K, while in the arc discharge region, it spans from 3000 to 12000 K. To investigate the effect of temperature on the decomposition characteristics of the gas mixture, molecular dynamics simulations of the model were conducted in the range of 300 K to 3200 K. CF_3_SO_2_F in CF_3_SO_2_F/CO_2_ begins to decompose at 1400 K, indicating that it remains stable in the presence of localized discharges as shown in Fig. 2a.

The buffer gas CO_2_ does not decompose between 1400 K and 2200 K, aligning with previous studies^24^. As the temperature increases from 1400 to 2200 K, the decomposition rate of CF_3_SO_2_F rises, with the decomposition ratio (the percentage of decomposed CF_3_SO_2_F) within 1000 ps increasing from 0.07 to 0.77, as illustrated in Fig. 2d. When the temperature reaches 3200 K (Fig. 2a), the decomposition ratio of CF_3_SO_2_F surges to 0.94 within 1000 ps, indicating rapid decomposition in the arc discharge channel.

We further investigate the effect of pressure on the decomposition behavior of CF_3_SO_2_F. The decomposition of CF_3_SO_2_F under pressures of 0.1, 0.3, and 0.5 MPa at 2200 K is shown in Fig. 2b. The decomposition rate and ratio of CF_3_SO_2_F increase with rising pressure. The decomposition ratio of CF_3_SO_2_F reaches to 0.87 and 0.94 at 0.3 and 0.5 MPa, respectively. This trend can be attributed to the increased pressure raising the concentration of molecules, thereby increasing the probability of intermolecular collisions and accelerating the decomposition rate and ratio of CF_3_SO_2_F.

To uncover the effect of gas mixing ratios on decomposition, the time evolution of CF_3_SO_2_F decomposition for different CF_3_SO_2_F/CO_2_ gas mixture systems at 2200 K is illustrated in Fig. 2c. Compared to 12%CF_3_SO_2_F/88%CO_2_ (0.77), the decomposition ratio of 14%CF_3_SO_2_F/86%CO_2_ (0.74) and 20%CF_3_SO_2_F/80%CO_2_ (0.64) within 1000 ps decrease sequentially. To address the potential influence of varying total system size, we employed two distinct modeling strategies. One strategy maintained a constant total number of molecules for all mixture ratios to directly control for system size effects. Conversely, the other strategy kept the number of CF_3_SO_2_F reactant molecules constant and varied only the number of buffer gas (N_2_ or CO_2_) molecules. The decomposition results from both approaches, analyzed over consistent time spans, are presented in Fig. S4. A similar result that the increased CF_3_SO_2_F mixing ratio would suppress the decomposition of the mixture can be observed. This may be primarily attributed to changes in molecular interactions and reaction kinetics. As the mixing ratio of CF_3_SO_2_F increases, the intermolecular forces, such as van der Waals forces and dipole-dipole interactions between CF_3_SO_2_F molecules^42,43^, become more significant. These interactions enhance the overall stability of the system, effectively suppressing the decomposition pathways. Moreover, in mixtures with a higher mixing ratio of CF_3_SO_2_F, a greater proportion of the collision energy is transferred into vibrational modes. Since CF_3_SO_2_F possesses more vibrational degrees of freedom than CO_2_ as shown in Tables S2 and S3, a higher proportion of the total energy is stored in these modes rather than in translation and rotation. This reduces the fraction of effective collisions where the energy in the translational and rotational degrees of freedom exceeds the activation barrier for decomposition, thereby lowering the reaction rate. The decomposition behavior of CF_3_SO_2_F remained consistent across different mixture ratios, regardless of the mixing gas model employed. The consistent decomposition behavior obtained from both the constant total number and constant CF_3_SO_2_F number approaches confirms the robustness of our findings.

As another commonly used buffering gas, N_2_ was systematically investigated to elucidate its influence on the thermal decomposition characteristics of CF_3_SO_2_F. Figures S5 and Fig. 2e, f present the decomposition trends of CF_3_SO_2_F/N_2_ mixtures under various temperatures, pressures, and mixing ratios conditions. Notably, the parametric dependencies observed in N_2_-based mixtures mirror those in CF_3_SO_2_F/CO_2_ systems, confirming the generalizability of our previous findings. However, the decomposition ratios of CF_3_SO_2_F in N_2_-buffered mixtures (ranging from 0.79 to 0.87) are notably lower than those observed in CO_2_-buffered systems (ranging from 0.87 to 0.94) at elevated pressures (0.3–0.5 MPa), as shown in Fig. 2d. This consistency was further verified by three separate simulations employing N_2_ or CO_2_ as buffer gases, respectively, as shown in Fig. S6. This statistically significant discrepancy indicates that N_2_ offers greater effectiveness in suppressing the thermal decomposition of CF_3_SO_2_F, highlighting its potential as a more efficient buffering gas in high-pressure applications. The differing effects of N_2_ and CO_2_ buffer gases on CF_3_SO_2_F decomposition stem from their distinct vibrational energy transfer efficiencies. Analysis of their vibrational spectra reveals that CO_2_, as a linear triatomic molecule, possesses low-frequency modes that effectively couple with the vibrational modes of excited CF_3_SO_2_F as shown in Tables S2 and S3. This facilitates resonant energy transfer during collisions, promoting greater energy accumulation in CF_3_SO_2_F and resulting in the observed higher decomposition yield in CO_2_ mixtures compared to N_2_, where such efficient vibrational coupling is absent.

### Main decomposition products distribution and evolution

**Fig. 3:**
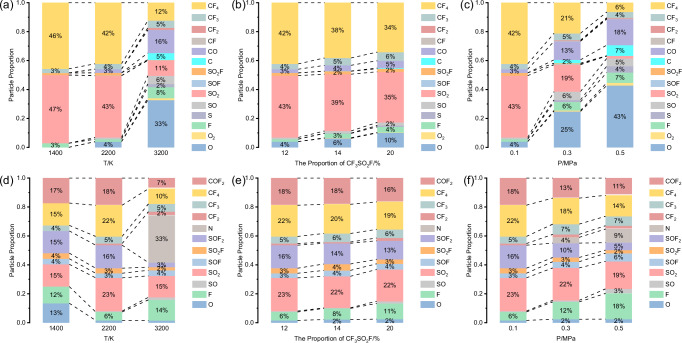
Comparative analysis of final decomposition products in CF_3_SO_2_F/CO_2_ and CF_3_SO_2_F/N_2_ mixtures. The relative concentration of CF_3_SO_2_F/CO_2_ final decomposition products **a** at 1400–3200 K, 12%CF_3_SO_2_F and 0.1 MPa, **b** at 2200 K, 12%CF_3_SO_2_F–20%CF_3_SO_2_F and 0.1 MPa, **c** at 2200 K, 12%CF_3_SO_2_F and 0.1–0.5 MPa. The relative concentration of CF_3_SO_2_F/N_2_ final decomposition products **d** at 1400–3200 K, 12%CF_3_SO_2_F and 0.1 MPa, **e** at 2200 K, 12%CF_3_SO_2_F–20%CF_3_SO_2_F and 0.1 MPa, **f** at 2200 K, 12%CF_3_SO_2_F and 0.1–0.5 MPa.

Figure S7a illustrates decomposition products of the CF_3_SO_2_F/CO_2_ mixture at temperatures ranging from 1400 to 3200 K. In this study, decomposition products are categorized as primary or secondary based on their formation mechanisms. Primary products, such as CF_4_ and SO_2_, are directly formed from the initial breakdown of CF_3_SO_2_F. Secondary products, such as SO and CF_2_, result from subsequent reactions involving intermediate species. In the analysis of decomposition products, CO_2_ originating from the buffer gas was systematically excluded to ensure that the reported product yields exclusively reflect new species formed from the decomposition of CF_3_SO_2_F and its subsequent reactions. The decomposition of the CF_3_SO_2_F/CO_2_ mixture begins at 1400 K. At this temperature, the extent of decomposition is limited, and the dominant reaction pathway yields CF_4_ and SO_2_. As the temperature increases to 2200 K, the number of primary decomposition products (CF_4_ and SO_2_) increase (Fig. S7a), while their relative concentrations decrease due to further decomposition into secondary products such as SO and CF_3_ (Fig. 3a).

As the temperature increases to 3200 K, the number of chemical reactions and byproducts in the system significantly increases. At this stage, the mixture transformed into CF_4_, CF_3_, CF_2_, CF, CO, C, SO_2_F, SOF, SO_2_, SO, S, F, O_2_, and O. This is attributed to the further breakdown of large molecular groups into smaller molecules. For instance, CF_4_ could decompose into CF_3_, CF_2_, CF, and C, while SO_2_ may decompose into SO and S. Consequently, O atoms become the most abundant molecular fragments in the system, originating from the decomposition of both the main insulating gas CF_3_SO_2_F and the buffer gas CO_2_.

The formation of C and S at high temperatures indicates that solid precipitation of these elements should be considered in the design of equipment insulation. The radial distribution function (RDF) provides quantitative insight into the spatial correlation between particles within the system. Figure S8b and c present RDF analyses of the CF_3_SO_2_F/CO_2_ mixture at 2200 and 3200 K, respectively. A pronounced weakening of the characteristic peaks corresponding to S–F, C–O, and S–O bonds is observed compared to the initial decomposition stage at 1400 K (Fig. S8a). This reduction directly reflects the progressive decomposition of CF_3_SO_2_F, CO_2_, and SO_2_ molecular species as temperature increases.

Figure S7d presents the final decomposition products of the CF_3_SO_2_F/N_2_ mixture across a temperature range of 1400–3200 K. In addition to CF_4_ and SO_2_, the primary decomposition products consist of COF_2_, SOF_2_ at lower temperatures which are absent in the CF_3_SO_2_F/CO_2_ mixture, suggesting distinct decomposition pathways between the two systems. Both the quantity and relative concentration of these major primary products increase as the temperature rises to 2200 K (Fig. 3d). The reaction complexity intensifies with a further increase in temperature (3200 K). N ≡ N triple bond dissociates, yielding atomic N, meanwhile the primary products (CF_4_, SO_2_, COF_2_, and SOF_2_) further decompose into secondary species such as CF_3_, CF_2_, SO_2_F, SOF, SO, N, F, and O. Consequently, the quantity and relative concentration of primary products decline as secondary products become dominant. Unlike the CF_3_SO_2_F/CO_2_ mixture, negligible formation of oxygen and solid residues (C and S) is observed, further demonstrating the mechanistic differences between the two mixture systems. From a thermodynamic perspective, the extreme temperatures in the simulation substantially increase the entropy of atomic and radical species. Although molecular nitrogen possesses an exceptionally strong N ≡ N bond, its dissociation into monatomic nitrogen becomes feasible under these conditions. The resulting free atoms are stabilized by the high-entropy environment, making the atomic state more favorable than recombination into less stable molecular products like nitrogen monoxide. Consequently, the buffer gas acts primarily through physical influences such as modulating collision frequency and energy distribution rather than as a key reactant in the core decomposition mechanism.

A comparison of the bond length distributions at 1400 K (Fig. S8d) with those at higher temperatures (Fig. S8e and f) reveals a clear temperature-dependent dissociation behavior, characterized by a broadening of the distribution and the emergence of a peak at longer distances corresponding to bond rupture. The C–S and S–F bonds, mainly associated with CF_3_SO_2_F and SO_2_F, the C–F bond, related to both CF_3_SO_2_F and CF_4_, and the S–O bond, primarily from SO_2_F, all exhibit noticeable weakening. This indicates the progressive decomposition of these molecular species.

To further investigate the effect of CF_3_SO_2_F mixing ratio on the decomposition products of CF_3_SO_2_F/CO_2_ and CF_3_SO_2_F/N_2_ mixtures, the final products quantity and the relative concentration of CF_3_SO_2_F mixtures with different CF_3_SO_2_F mixing ratios at 2200 K were simulated as shown in Figures S7b, e, and 3b, e. The number of decomposition products decreases as the CF_3_SO_2_F mixing ratio in the system increases, consistent with the CF_3_SO_2_F decomposition evolution curves (*vs*. time) at different mixing ratios in Fig. 2c and f. Using CF_3_SO_2_F/CO_2_ mixtures as an example, the SO_2_ yield exhibits an inverse relationship with the CF_3_SO_2_F mixing ratio. Specifically, the 12%CF_3_SO_2_F/88%CO_2_ mixture generates 358 SO_2_ molecules after 1000 ps under 2200 K, whereas the 20%CF_3_SO_2_F/80%CO_2_ system produces only 280 SO_2_ molecules. Moreover, as the mixing ratio of CF_3_SO_2_F in the system increases (Fig. 3b), the relative concentration of CF_4_ and SO_2_ decreases, while the relative concentration of secondary products increases slightly. This trend aligns with the evolution of the final products quantity and relative concentration for mixture systems maintaining constant total number of molecules (Fig. S9), demonstrating the robustness of the results.

To examine the pressure dependence of CF_3_SO_2_F decomposition products, simulations were conducted for CF_3_SO_2_F/CO_2_ and CF_3_SO_2_F/N_2_ mixtures at 2200 K across a pressure range of 0.1–0.5 MPa. Figures S7c and 3c illustrate the evolution of final products and product relative concentration in the CF_3_SO_2_F/CO_2_ system. It can be seen that elevated pressure markedly accelerates reactions, promoting the transformation of primary products (CF_4_ and SO_2_) into smaller fragments, including SO, CF_3_, and monoatomic species (C, S, F, and O). At 0.5 MPa, CO_2_ undergoes substantial dissociation, yielding CO and O. The resulting [CO]/[CO_2_] ratio of 0.27 in our simulations shows remarkable agreement with the value of 0.26 reported in previous studies^24^. Furthermore, the O atom is the most abundant single product (1714 particles, 43% of the total). A parallel trend is observed for CF_3_SO_2_F/N_2_ (Figs. 3f, S7f), where high pressure similarly drives further fragmentation of primary products, though with comparatively lower decomposition yields.

### Decomposition mechanism of CF_3_SO_2_F

**Fig. 4:**
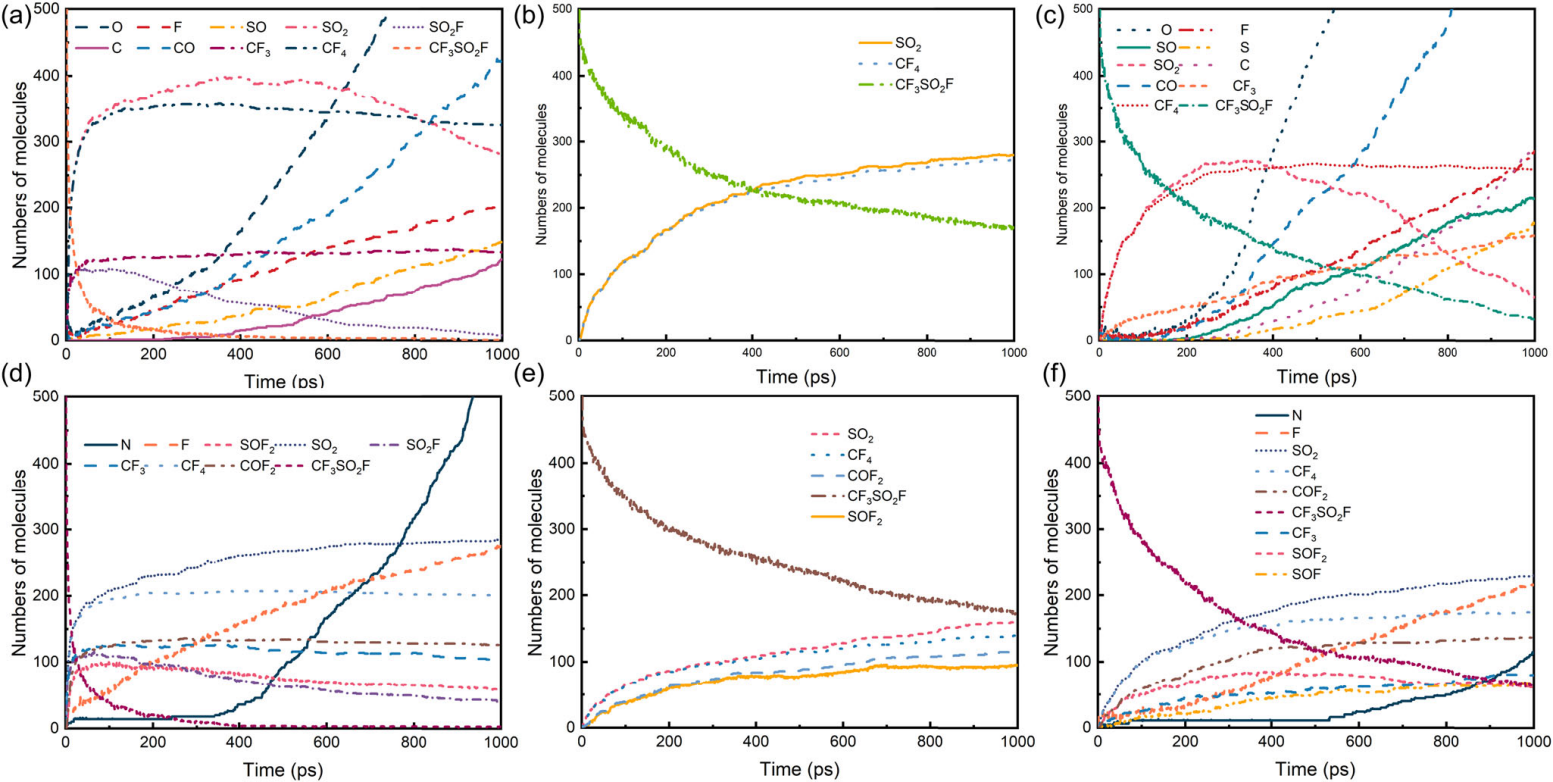
Decomposition product dynamics in CF_3_SO_2_F mixtures. Time evolution of major decomposition products in CF_3_SO_2_F/CO_2_ mixtures under different conditions: **a** at 3200 K, 12%CF_3_SO_2_F and 0.1 MPa, **b** at 2200 K, 20%CF_3_SO_2_F and 0.1 MPa, **c** at 2200 K, 12%CF_3_SO_2_F and 0.5 MPa. Time evolution of major decomposition products in CF_3_SO_2_F/N_2_ mixtures under different conditions: **d** at 3200 K, 12%CF_3_SO_2_F and 0.1 MPa, **e** at 2200 K, 20%CF_3_SO_2_F and 0.1 MPa, **f** at 2200 K, 12%CF_3_SO_2_F and 0.5 MPa.

To elucidate the thermal decomposition mechanisms of CF_3_SO_2_F/CO_2_ mixtures under varying conditions, we examined the temporal evolution of decomposition products. Both product yields and reaction rates showed strong positive correlations with temperature. Notably, the reaction profiles at 3200 K (Fig. 4a) were significantly steeper than those at 2200 K (Fig. S10), indicating an acceleration of the reaction rate at elevated temperatures. Notably, the variation of SO_2_ number reveals distinct reaction process: (1) an initial rapid accumulation phase (0–100 ps) characterized by a steep number increase, (2) an intermediate transition phase (100–500 ps) showing progressively slower accumulation, and (3) a final depletion phase (> 500 ps) exhibiting accelerated number decline. This behavior reflects rapid CF_3_SO_2_F decomposition initially (CF_3_SO_2_F → CF_4_ + SO_2_), producing SO_2_ faster than its decomposition. As CF_3_SO_2_F depletes, the formation rate of SO_2_ slows. However, the concurrent increase in the concentrations of SO_2_ molecules promotes its subsequent decomposition reactions, leading to its net consumption under these conditions. The presence of the product SO_2_F suggests that a high-temperature decomposition pathway involving direct C–S bond cleavage through intense molecular vibrations, yielding CF_3_ and SO_2_F for subsequent decomposition.

The CF_3_SO_2_F mixing ratio significantly influenced decomposition dynamics (Fig. 4b). Increasing the CF_3_SO_2_F mixing ratio in the mixture elevated the gas density (Table 1) and suppressed CF_3_SO_2_F decomposition, leading to a decrease in the yields of primary products (CF_4_ and SO_2_). Both reaction rate and product species exhibited a positive pressure dependence (Fig. 4c). At higher pressures, in addition to the primary products CF_4_ and SO_2_, small molecular fragments such as CF_3_, SO, F, O, S, and C are generated in significantly greater quantities, which suggests that higher pressures promote more extensive molecular dissociation of CF_3_SO_2_F.

**Fig. 5:**
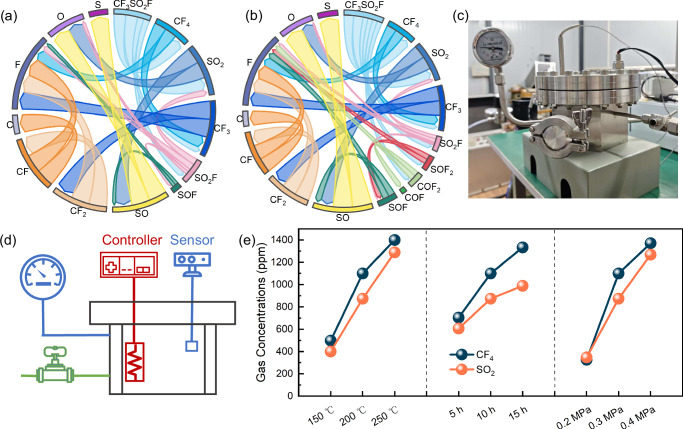
Decomposition mechanisms and experimental validation. Decomposition mechanisms of CF_3_SO_2_F (**a**) in the CF_3_SO_2_F/CO_2_ mixed gas and (**b**) in the CF_3_SO_2_F/N_2_ mixed gas. **c** The physical setup and **d** schematic diagrams of the thermal decomposition test platform. **e** Measured gas concentrations of 14%CF_3_SO_2_F/86%N_2_ thermal decomposition under different conditions.

The decomposition of CF_3_SO_2_F is the dominant factor governing the primary reaction pathways and product distribution in CF_3_SO_2_F/CO_2_ mixtures, as shown in Fig. 5a and Table S4. Trajectory simulations reveal that the net reaction CF_3_SO_2_F → CF_4_ + SO_2_ proceeds via two temperature-dependent pathways rather than a single concerted step. The primary product formation (CF_4_ + SO_2_) occurs through a concerted mechanism where C–S bond cleavage and fluorine transfer from SO_2_F to CF_3_ proceed simultaneously at temperatures above 1400 K as shown in Fig. S11. This concerted process, mediated by a transition state, directly yields CF_4_ and SO_2_, which is consistent with previous studies^15^. At temperatures exceeding 2200 K, direct C–S bond rupture becomes increasingly prevalent, producing CF_3_ and SO_2_F radicals. SO_2_F then decomposes primarily through S–F bond cleavage (yielding SO_2_ + F) with minor S–O cleavage (producing SOF + O). The resulting SOF decomposes further to SO and F. Primary products undergo progressive defluorination and deoxygenation, ultimately generating atomic species.

The CF_3_SO_2_F/N_2_ system demonstrates decomposition mechanisms analogous to CF_3_SO_2_F/CO_2_ while displaying distinct kinetic and product characteristics. At lower temperatures, CF_3_SO_2_F decomposition with N_2_ existing proceeds predominantly through two competing pathways: (1) direct formation of primary product of SO_2_ and CF_4_ (CF_3_SO_2_F → CF_4_ + SO_2_), and (2) direct formation of secondary product of COF_2_ and SOF_2_ (CF_3_SO_2_F → COF_2_ + SOF_2_) as shown in Fig. S12. Upon increasing the temperature to 3200 K, an additional pathway emerges involving direct C–S bond cleavage to yield CF_3_ and SO_2_F radicals (Fig. 4d), mirroring the behavior observed in CO_2_-based mixtures. Notably, in the N_2_ buffer gas, variations in the CF_3_SO_2_F mixing ratio show minimal impact on the fundamental reaction pathways, as evidenced by the consistent final product species across different mixture ratios (Figs. 4e and S12), even though the absolute product yields vary with the initial reactant amount. However, the system exhibits significantly reduced product diversity and slower reaction kinetics under elevated pressures (Fig. 4f), confirming the superior efficacy of N_2_ as a decomposition inhibitor compared to CO_2_. The distinct decomposition mechanism of CF_3_SO_2_F/N_2_ (Figs. 5b and S13) primarily differs through the inclusion of the COF_2_/SOF_2_ formation pathway. The absence of the COF_2_/SOF_2_ formation pathway in the CO_2_ system contributes to the observed differences in product profiles and decomposition rates between the two mixtures.

### Experimental validation of CF_3_SO_2_F decomposition

To validate the simulation results, an overheating decomposition experiment was performed to investigate the thermal decomposition characteristics of 14%CF_3_SO_2_F/86%N_2_ gas mixtures. The experiments were systematically conducted under varying temperatures (150–250 °C in 50 °C increments), overheating duration (5h–15 h in 5 h increments), and gas pressures (0.2–0.4 MPa in 0.1 MPa increments), corresponding to the typical operational range of high voltage gas insulated equipment.

Following each test, the residual gas composition in the reaction chamber was sampled and analyzed. The GC-MS test result of the decomposition products of 14%CF_3_SO_2_F/86%N_2_ at 200 °C for 15 h is illustrated in Fig. S14. CF_4_ and SO_2_ were identified as the dominant decomposition products. Consequently, their yield variations under different conditions were systematically analyzed. As shown in Fig. 5e, the quantitative results demonstrate that CF_4_ and SO_2_ are the primary decomposition products with comparable concentrations, which aligns with the key predictions of the computational model. Furthermore, the observed consistent increase in their yields with rising temperature, extended reaction time, and elevated pressure firmly establishes the validity of the proposed decomposition mechanism. Furthermore, the radicals generated during decomposition exhibit a low tendency to recombine into CF_3_SO_2_F, further confirming the irreversible decomposition process.

## Discussion

In summary, the decomposition mechanism of CF_3_SO_2_F/CO_2_ and CF_3_SO_2_F/N_2_ gas mixture at different temperatures, gas mixing ratios, and pressures was theoretically investigated and experimentally verified by deep learning potential training and molecular dynamics simulation. The results show that the decomposition of CF_3_SO_2_F/CO_2_ gas mixture starts at 1400 K under the time scale of 1000 ps, and the primary decomposition products are CF_4_ and SO_2_. When the temperature increases to 3200 K, CF_3_SO_2_F will decompose rapidly in the arc discharge channel, and the decomposition ratio of CF_3_SO_2_F reaches 0.94 within 1000 ps and decomposes to CF_4_, CF_3_, CF_2_, CF, CO, C, SO_2_F, SOF, SO_2_, SO, S, F, O_2_, O. At lower temperatures, the CF_3_SO_2_F molecule tends to cleave the C–S bond, with the F atom on SO_2_F transferring to CF_3_ during the bond breakage, ultimately forming CF_4_ and SO_2_. In contrast, at higher temperatures, increased molecular vibrations cause the C–S bond to break more directly, leading to the formation of CF_3_ and SO_2_F, which then undergo further decomposition. Furthermore, a higher initial concentration of CF_3_SO_2_F increases the absolute number of decomposition events. The accelerated accumulation of primary products such as CF_4_ and SO_2_ favors their own secondary reactions. The initial decomposition rate of CF_3_SO_2_F is significantly promoted by elevated system pressure due to the corresponding increase in molecular collision frequency. For engineering applications, this finding highlights the essential need for rigorous condition monitoring of electrical equipment to prevent significant CF_3_SO_2_F decomposition due to localized overheating, which would degrade its electrical insulation performance.

The primary decomposition pathways of CF_3_SO_2_F/N_2_ and CF_3_SO_2_F/CO_2_ mixtures are similar, except for an additional reaction in the latter system (CF_3_SO_2_F → COF_2_ + SOF_2_). The effects of temperature, gas composition, and pressure on the decomposition behavior of CF_3_SO_2_F in N_2_ closely resemble those observed in CO_2_. However, under high-pressure conditions, N_2_ acts as an inert buffer gas and more effectively suppresses the decomposition of CF_3_SO_2_F compared to CO_2_.

While CF_3_SO_2_F is proposed as an environmentally friendly alternative to SF_6_, the potential impact of its decomposition products should be rigorously assessed. Our simulations identify CF_4_ and COF_2_ as key decomposition species. It is critical to note that while the GWP of CF_4_ (7390) is significantly lower than that of SF_6_ (23500), it remains higher than that of carbon dioxide, with an atmospheric lifetime exceeding 50,000 years. Furthermore, COF_2_ is a highly toxic compound, posing potential safety hazards in the event of insulator failure and gas release. Our results, however, provide crucial insights for mitigating these risks. The formation of both CF_4_ and COF_2_ exhibits a strong dependence on temperature and the buffer gas environment. Specifically, using N_2_ as a buffer gas can suppress the formation of these harmful byproducts, potentially serving as an effective strategy to minimize environmental and safety risks. Therefore, the viability of CF_3_SO_2_F as an SF_6_ replacement hinges not only on its intrinsic properties but also on engineering controls that optimize operating conditions to limit the formation of deleterious by-products. This molecular-level understanding directly informs the development of safer, next-generation gas-insulated equipment aligned with global decarbonization goals.

In conclusion, this study identifies the major decomposition products and elucidates the primary reaction mechanisms of CF_3_SO_2_F under high-temperature and high-pressure conditions. The established computational framework provides a foundation that can inform future assessments of environmental impact and guide the development of safer CF_3_SO_2_F-based insulation technologies.

## Methods

### AIMD calculations setup

AIMD simulations were executed to construct the training dataset for machine learning. All calculations were carried out in the Vienna ab initio simulation packages (VASP)^44,45^. Ion-electron interactions were modeled with the Projector Augmented Wave (PAW) method^46^. Using the revised Perdew−Burke−Ernzerhof functional for describing electronic exchange and correlations, DFT calculations were executed employing the generalized gradient approximation method^47^. The plane-wave basis was applied with a cutoff energy of 520 eV. The convergence criteria for the electronic energy and structural relaxation were set to 10^–6^ eV and 0.01 eV/Å, respectively. AIMD simulations were performed in the canonical ensemble (NVT) with periodic boundary conditions and a time step of 0.5 fs to obtain configurations, energies, forces, and virials over a wide range of temperatures for the CF_3_SO_2_F gas mixture with three CF_3_SO_2_F molecules and six buffer gas molecules in the box. In the NVT simulations, the Nosé-Hoover thermostat was employed to maintain isothermal conditions at 30–3200 K for 20 ps. Molecular species were identified from the MD trajectories using a topology analysis algorithm based on interatomic distances and bond orders. This method allows for the dynamic tracking of bond formation and dissociation, enabling continuous identification of all chemical species throughout the simulation. The analysis script is available in the associated GitHub repository. Details of DP-GEN, DLP training, and MD setup were listed in the Supplementary Methods section of the Supporting Information.

### Thermal decomposition characteristics test platform

The experimental platform for investigating thermal decomposition characteristics comprises three main components: (1) a high-temperature test chamber, (2) a temperature control system, and (3) a DC power supply. The stainless steel test chamber (340 L grade, 10 L capacity) operates within a pressure range of 0–0.4 MPa. The thermal decomposition tests were conducted using a custom experimental platform, with the physical setup and schematic diagrams shown in Fig. 5c, d. The system pressure was monitored by a high-precision digital barometer connected to the chamber. A temperature control system, integrating electromagnetic relays with sensors and controllers, maintained precise thermal conditions. A DC power supply energized thermocouples to simulate the localized overheating faults typical in gas-insulated equipment. The gas composition and relative concentration of CF_3_SO_2_F mixed gas after the test were analyzed by gas chromatography–mass spectrometry (GC–MS) equipped with a GS-GASPRO column (60 m). The inlet temperature was maintained at 100 °C with a split ratio of 20:1. Helium carrier gas was used at a constant flow mode with a total flow of 57.0 mL/min, a column flow of 2.43 mL/min, and a linear velocity of 39.7 cm/s. The oven temperature program was as follows: held at 40 °C for 1 min, ramped to 120 °C at 7 °C/min, and finally held at 120 °C for 6 min. The MS ion source and transfer line temperatures were both set to 200 °C. Species identification was achieved by matching the acquired mass spectra and retention times against reference standards.

## Supplementary information

Transparent Peer Review file

Support Information

Description of Additional Supplementary Files

Supplementary Data 1

## Data Availability

All data supporting the findings of this study are available within the article and its Supplementary Information. Source data are available as Supplementary Data 1.

## Code availability

The DLP models are available on GitHub at https://github.com/LZYUCL/DLP_CF3SO2F and archived on Zenodo at 10.5281/zenodo.17731042.
