# Supplementary material for: Probing the thermal decomposition mechanism of CF3SO2F by deep learning molecular dynamics
Source: Commun Chem. 2025 Dec 19;9:40. doi: 10.1038/s42004-025-01847-x (PMC12824197; doi:10.1038/s42004-025-01847-x)
Supplement: Supplementary file 2 — Support Information [file 42004_2025_1847_MOESM2_ESM.pdf]

Supporting Information

Probing the thermal decomposition mechanism of CF_3SO_2F by deep learning molecular dynamics

Anyang Wang[1], Zeyuan Li[2], Shubo Ren[1], Xue Ke[1], Xuhao Wan[1], Rong Han[1], Xianglian Yan[3], Wen Wang[3], Yu Zheng[1], Yuzheng Guo[1,2*], Jun Wang[1*]*

[1]*School of Power and Mechanical Engineering, Wuhan University, Wuhan 430072, China*

[2]*School of Electrical Engineering and Automation, Wuhan University, Wuhan 430072, China*

[3]*High Voltage Department, China Electric Power Research Institute, Beijing, China*

***Corresponding author**:

Prof. Yu Zheng (zywhuee@whu.edu.cn)

Prof. Yuzheng Guo (yguo@whu.edu.cn)

Prof. Jun Wang (junwangwhu@whu.edu.cn)

Supplementary Methods

DP-GEN setup. The concurrent learning procedure DP-GEN was employed to refine the configuration space of the CF_3SO_2F gas mixture iteratively, which has been shown to accurately reproduce the interatomic forces and energies predicted by *ab initio* calculations.[1] In the DP-GEN workflow, each iterations consisting of three processes: training, exploration, and labeling. The initial iteration used the AIMD dataset to train the DLP model. During the exploration process, the open-source code Large-scale Atomic/Molecular Massively Parallel Simulator (LAMMPS)[2] was used for NVT iterations with the Nosé-Hoover thermostat. The maximum standard deviation of the atomic forces predicted by the model ensemble, ϵ, served as the error indicator.[1] Configurations with model deviation 0.05 eV/Å $\leq \epsilon < 0.50$ eV/Å were selected for the labeling process. In the labeling process, VASP performed DFT calculations on these candidate structures, adhering to the same force and energy convergence criteria as those used in the AIMD calculations. As a generalized gradient approximation (GGA), PBE is known to underestimate reaction barriers due to its limitations in modeling medium- and long-range correlation. Nonetheless, it was selected for its computational efficiency and proven ability to qualitatively describe the bond dissociations and radical interactions relevant to CF_3SO_2F decomposition.[3,4] Benchmark calculations against B3LYP data confirm that PBE correctly reproduces the potential energy surfaces of the CF_3SO_2F molecule (including CF_3−SO_2F, F−CF_2SO_2F, F−CF_3SO_2, and O−CF_3SOF bond dissociation energies) as shown in **Figure S15**, validating its use for identifying dominant mechanisms. The new dataset was generated from these DFT calculations, and the initial dataset was used to train a new DLP model for subsequent exploration processes. Detailed training parameters can be found in the DLP training setup. The DP-GEN process culminated in a comprehensive dataset of the CF_3SO_2F gas mixture configurations across a wide range of temperatures.

DLP training setup. The DeePMD-kit package was utilized to train the DLP model and to interface it with LAMMPS to model interactions in the CF_3SO_2F gas mixture.[5] We selected the 'se_e2_a' descriptor type, employing a cutoff radius of 10.00

Å to capture interatomic interactions. The descriptor used a neural network architecture with layers of 25, 50, and 100 neurons, respectively. The fitting network consisted of three layers, with 250, 250, and 250 neurons in each layer, ensuring effective learning of the complex model. An exponentially decaying learning rate was employed, decreasing from an initial value of 0.001 to 3.51×10^{-8}, with different loss function weights assigned for energy, force, and virial optimization. The DLP model was retrained with a cutoff radius of 10.00 Å.

MD setup. We used LAMMPS for MD simulations,[2] integrated with our trained DLP model. We performed simulations at different temperatures (1400 K~3200 K) with pressures of 0.1~0.5 MPa. The initial structures were equilibrated for 5 ps in the NPT ensemble and for 10 ps in the NVT ensemble at 1000 K to minimize the system. We used a Nose-Hoover thermostat, with a temperature dumping parameter of 0.1 ps, and a timestep of 0.5 fs to explore the decomposition process of the CF_3SO_2F gas mixture. The Open Visualization Tool (OVITO) was used to analyze the thermal decomposition processes in the MD simulations, specifically the Radial Distribution Function.[6]

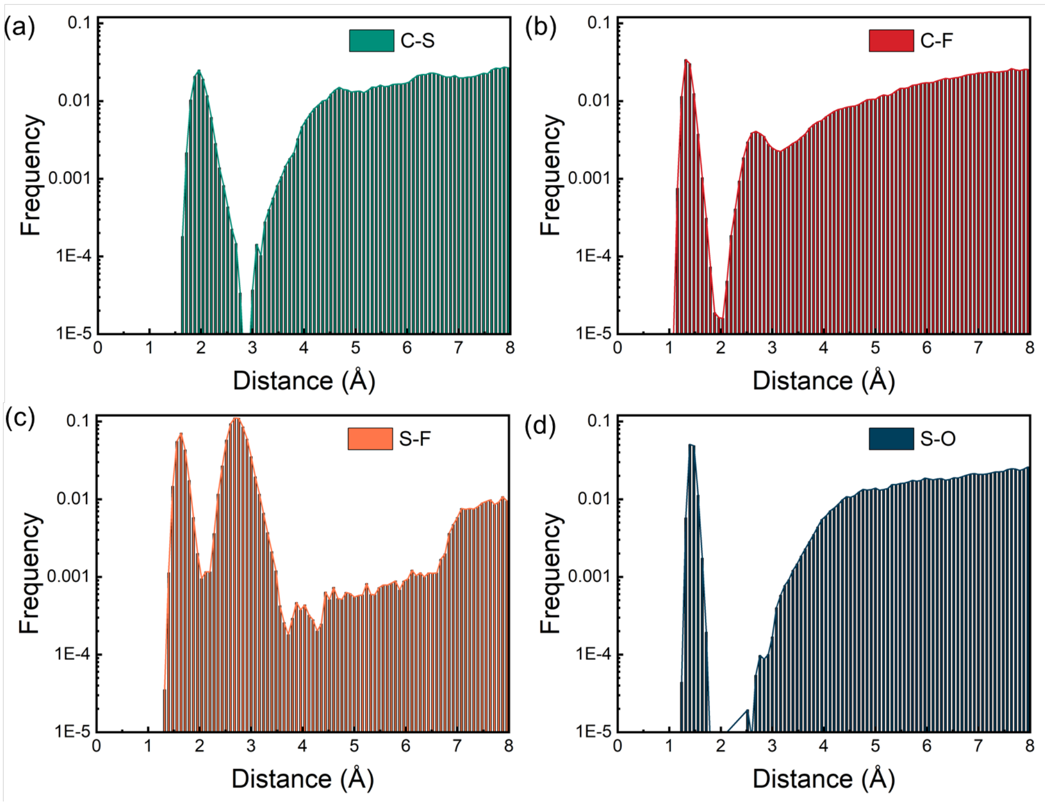

Figure S1. The frequency of initial dataset configurations based on AIMD trajectories.

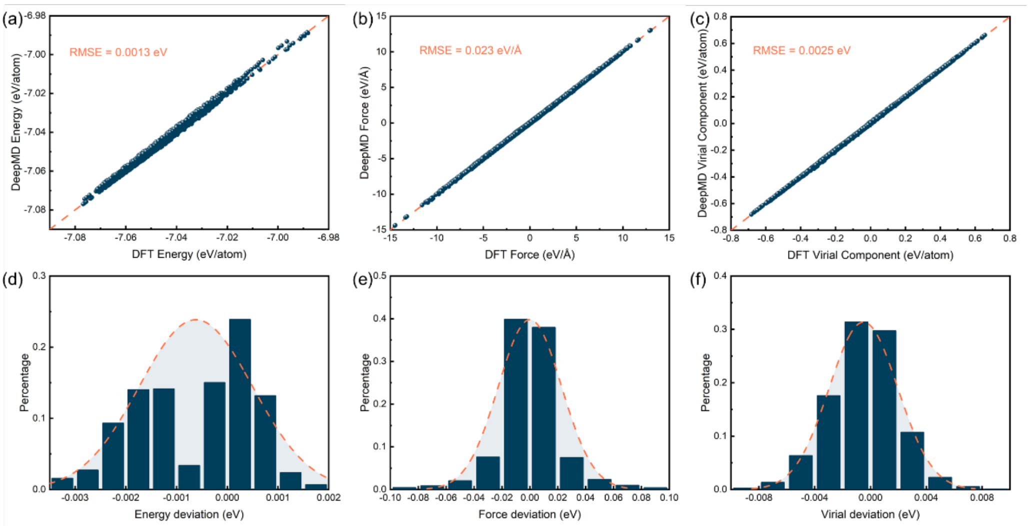

Figure S2. Scatter plot for (a) total energies, (b) forces on the *x*, *y*, and *z* axes, and (c) virials computed at the DFT level and predicted by the DLP. (d) Energy, (e) force and (f) virial deviation distributions for DLP.

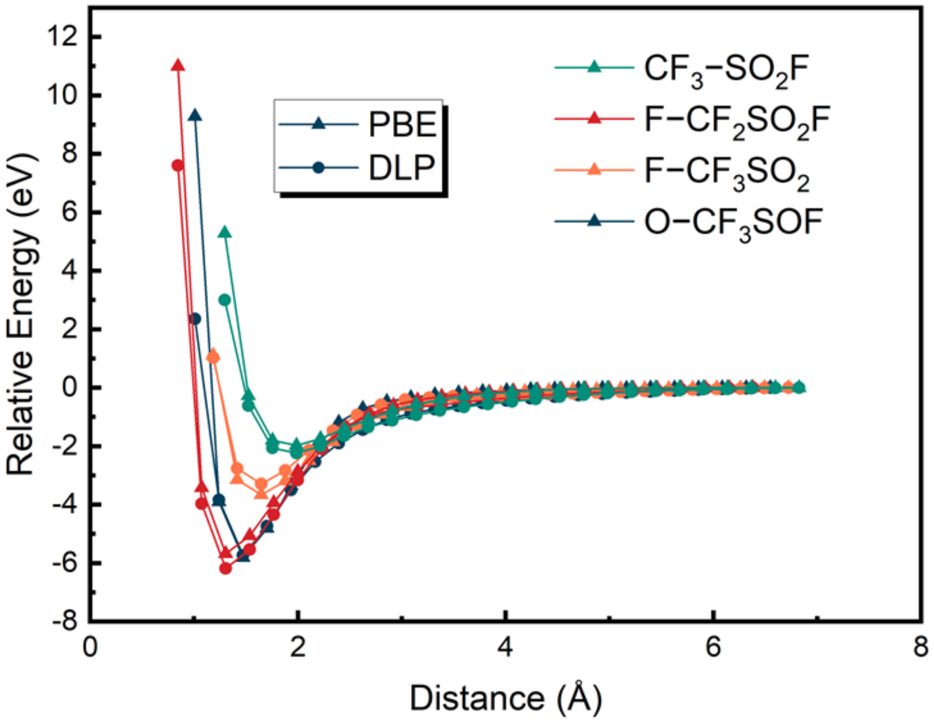

Figure S3. The potential energy surfaces of the CF_3SO_2F molecule with changing $CF_3{-}SO_2F$, $F{-}CF_2SO_2F$, $F{-}CF_3SO_2$, and $O{-}CF_3SOF$ bond lengths.

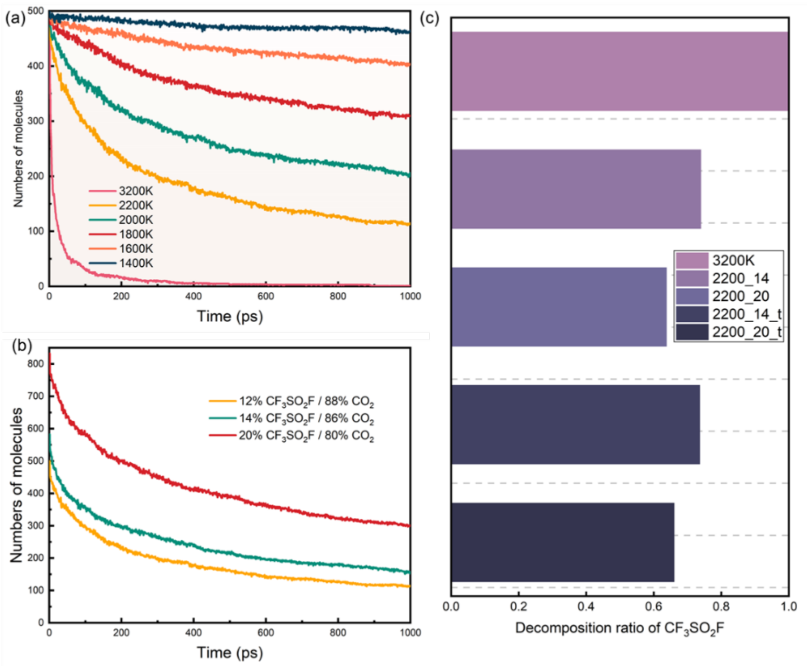

Figure S4. (a) Time evolution of CF_3SO_2F decomposition at 1400-3200 K in the CF_3SO_2F/CO_2 mixed gas. (b) Time evolution of CF_3SO_2F decomposition at 2200 K with different content in the CF_3SO_2F/CO_2 mixed gas. (d) Final decomposition ratio of CF_3SO_2F under different conditions within 1000 ps in the CF_3SO_2F/CO_2 mixed gas.

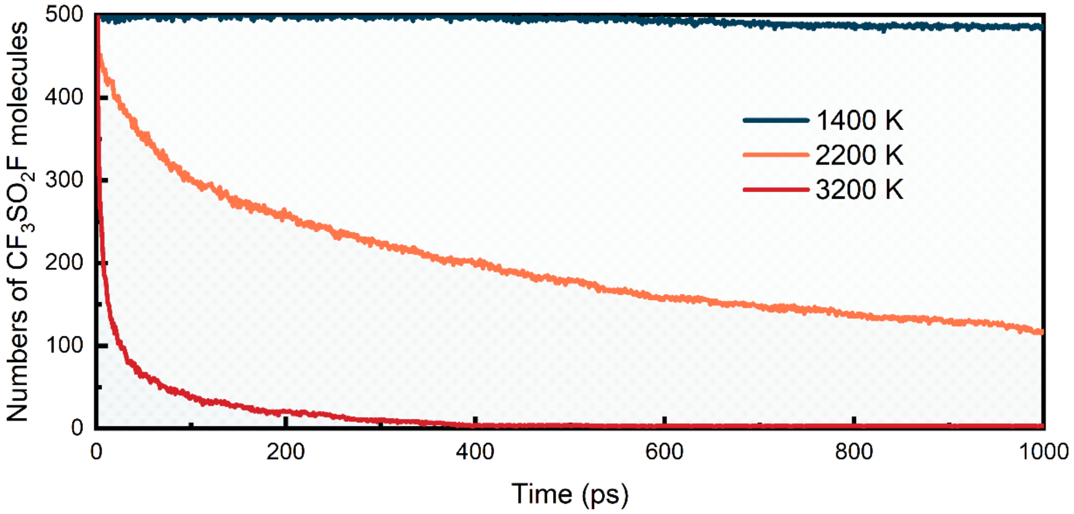

Figure S5. Time evolution of CF_3SO_2F decomposition at 1400-3200 K in the CF_3SO_2F/N_2 mixed gas.

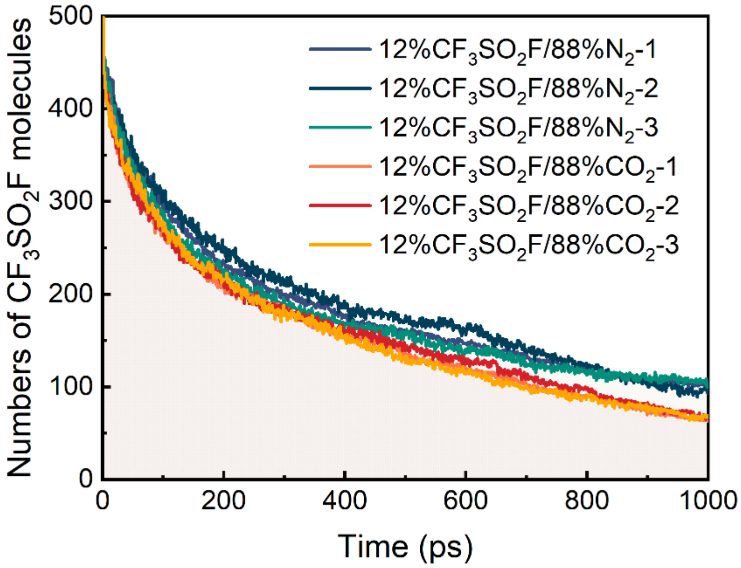

Figure S6. Time evolution of CF_3SO_2F decomposition in CO_2 and N_2 buffer gases.

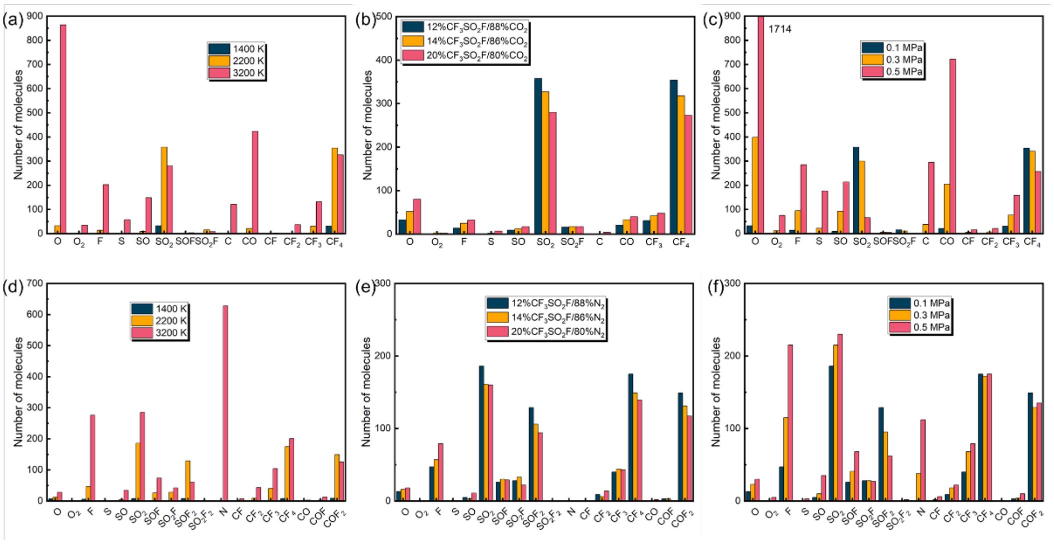

Figure S7. The final amount of CF_3SO_2F/CO_2 decomposition products (a) at 1400–3200 K, 12%CF_3SO_2F and 0.1 MPa, (b) at 2200 K, 12%CF_3SO_2F–20%CF_3SO_2F and 0.1 MPa, (c) at 2200 K, 12%CF_3SO_2F and 0.1–0.5MPa. The final amount of CF_3SO_2F/N_2 decomposition products (d) at 1400–3200 K, 12%CF_3SO_2F and 0.1 MPa, (e) at 2200 K, 12%CF_3SO_2F–20%CF_3SO_2F and 0.1 MPa, (f) at 2200 K, 12%CF_3SO_2F and 0.1–0.5MPa.

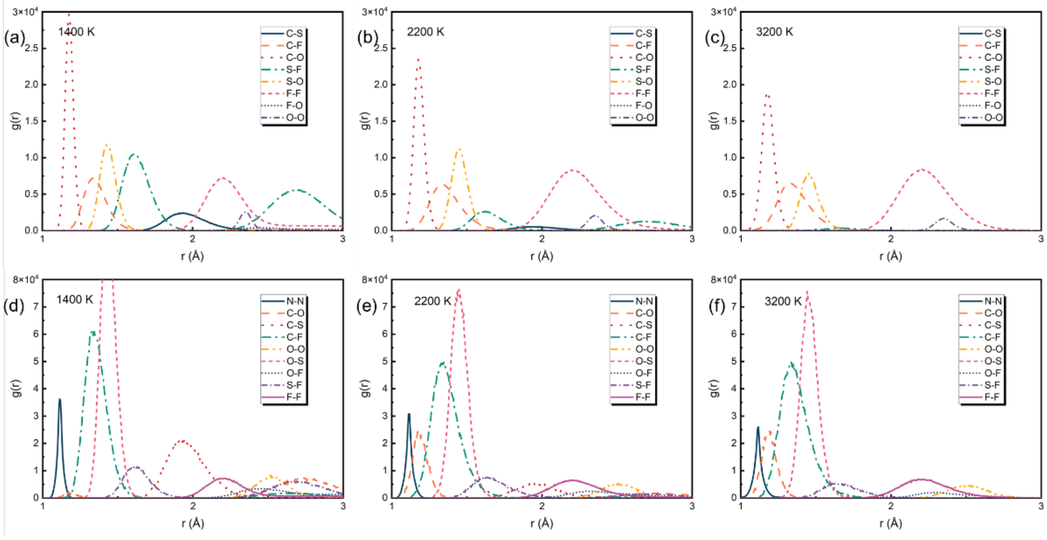

Figure S8. Radial distribution function g(r) of CF_3SO_2F/CO_2 at (a) 1400, (b) 2200, and (c) 3200 K. Radial distribution function g(r) of CF_3SO_2F/N_2 at (d) 1400 K, (e) 2200 K, and (f) 3200 K.

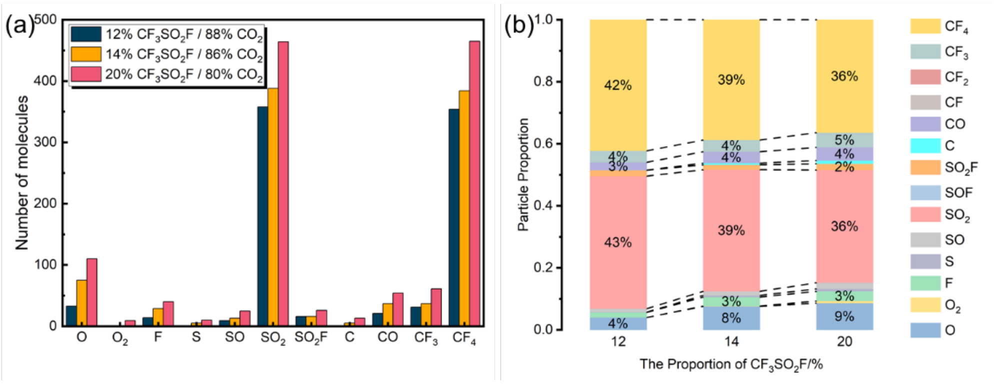

Figure S9. (a) The final amount of CF_3SO_2F/CO_2 decomposition products with $12\%CF_3SO_2F$-$20\%CF_3SO_2F$. (b) Evolution of CF_3SO_2F/CO_2 final decomposition products with $12\%CF_3SO_2F$-$20\%CF_3SO_2F$. The simulation box used is the total number of molecules keeps constant.

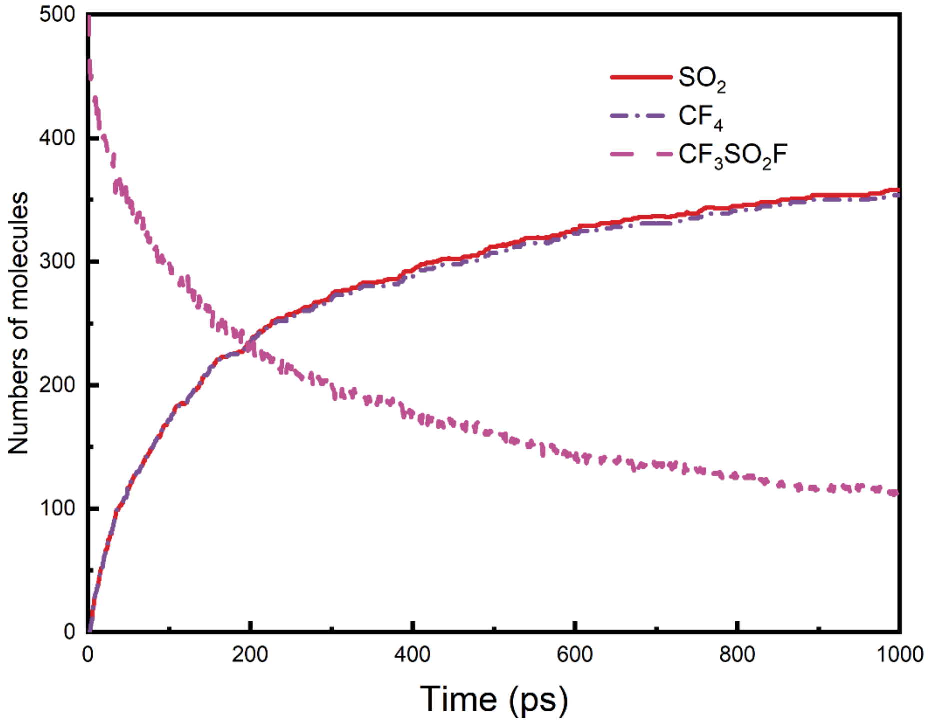

Figure S10. Time evolution of CF_3SO_2F/CO_2 decomposition products at 2200 K.

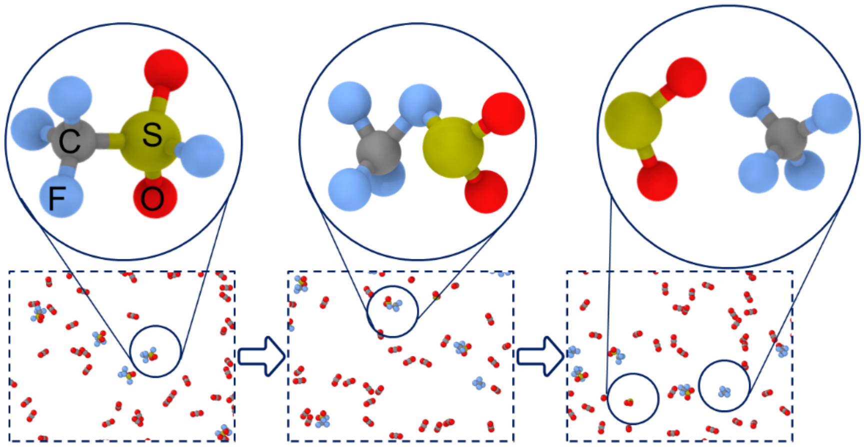

Figure S11. CF_3SO_2F decomposition at moderate temperatures.

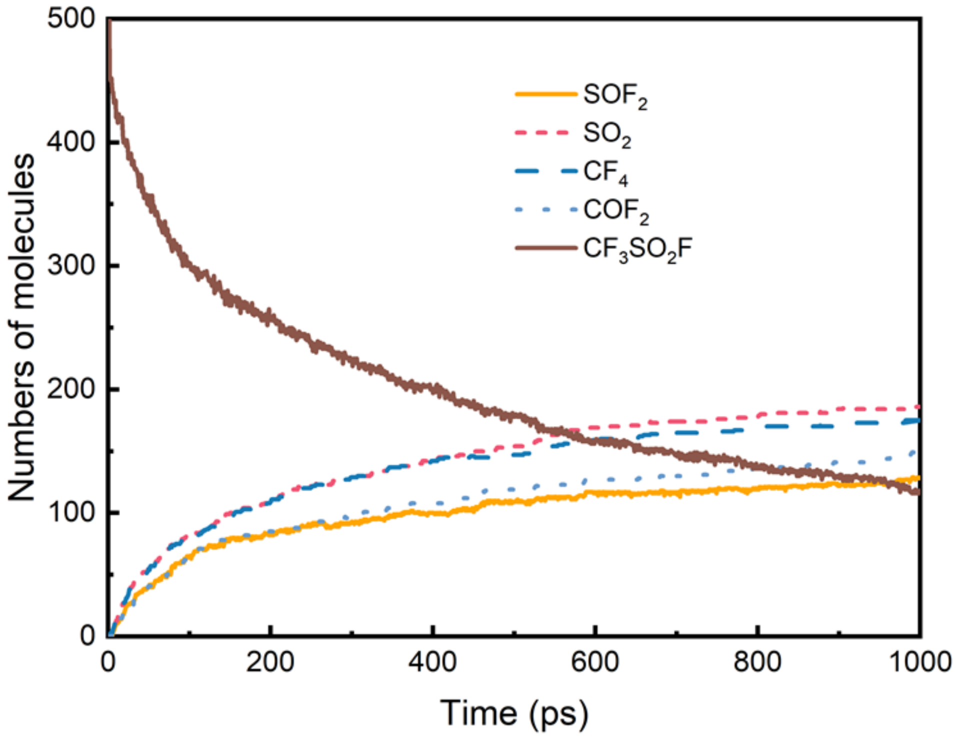

Figure S12. (a) Time evolution of CF_3SO_2F/N_2 decomposition products at 2200 K.

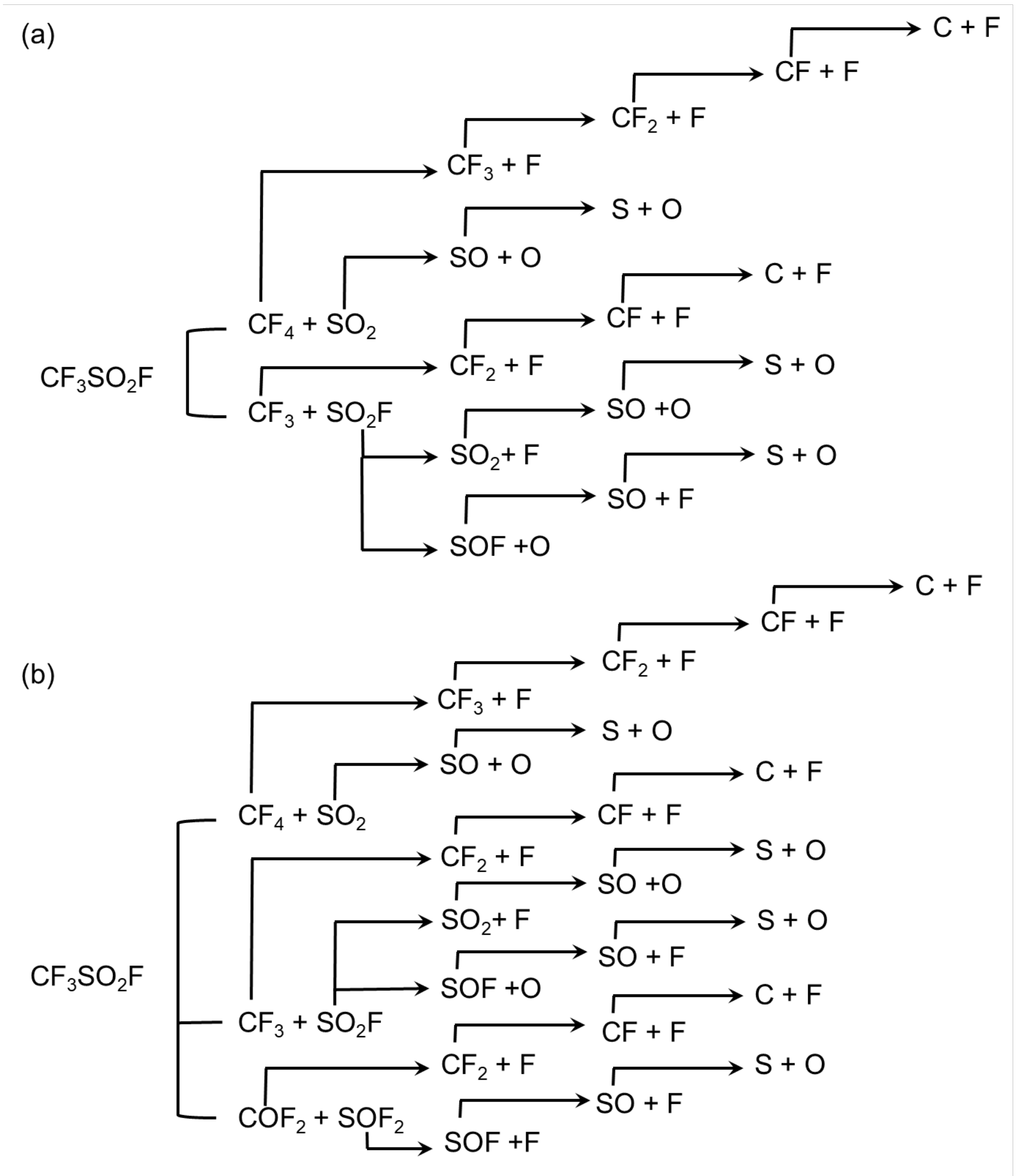

Figure S13. The decomposition pathways of CF_3SO_2F in (a) CO_2 and (b) N_2 buffer gases.

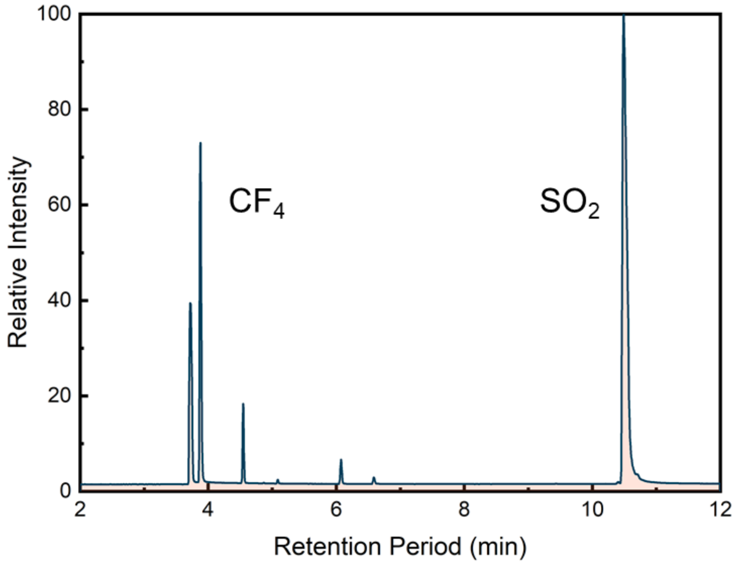

Figure S14. GC–MS results of 14%CF_3SO_2F/86%N_2 at 200 ℃.

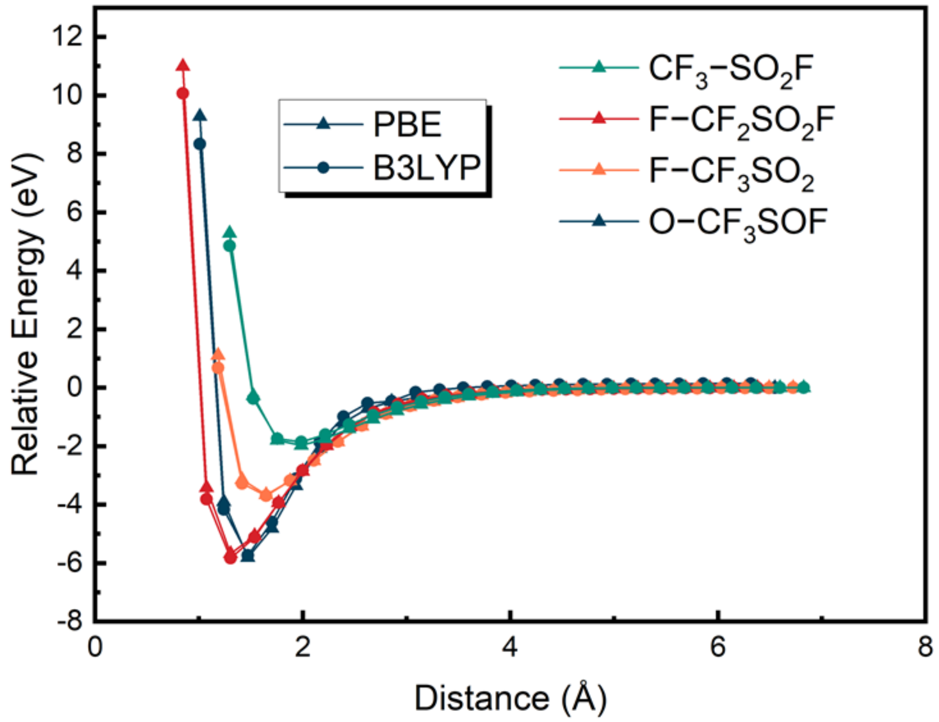

Figure S15. The potential energy surfaces of the CF_3SO_2F molecule with changing CF_3−SO_2F, F−CF_2SO_2F, F−CF_3SO_2, and O−CF_3SOF bond lengths.

Table S1. Parameters of the simulated CF_3SO_2F/N_2 systems. The pressure listed is the initial ideal gas pressure calculated from the given number of molecules in the simulation cell at 300 K.

No	P	ratio	CF_3SO_2F	N_2	atoms	Density (g/cm^3)	box length/Å
1	0.1MPa	0.20	500	2000	10000	0.002158	466.6134
2	0.1MPa	0.14	500	3070	10140	0.001854	525.5068
3	0.1MPa	0.12	500	3666	11332	0.001753	553.2428
4	0.3MPa	0.12	500	3666	11332	0.005258	383.5971
5	0.5MPa	0.12	500	3666	11332	0.008764	323.5384

Table S2. Vibrational Frequencies and Modes of CO_2 and N_2.

No.	Frequencies (cm^{-1})	Modes	No.	Frequencies (cm^{-1})	Modes
1	651.85	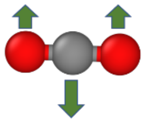	1	2370.23	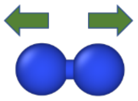
2	664.52	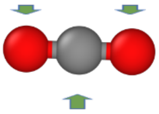			
3	1328.76	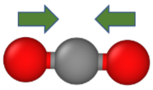			
4	2372.61	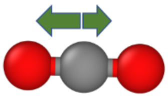			

Table S3. Vibrational Frequencies and Modes of the CF_3SO_2F.

No.	Frequencies (cm^{-1})	Modes	No.	Frequencies (cm^{-1})	Modes
1	49.47	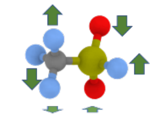	10	531.56	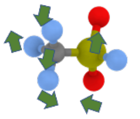
2	167.90	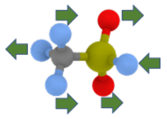	11	558.00	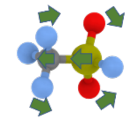
3	172.28	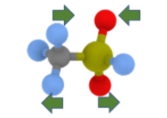	12	706.19	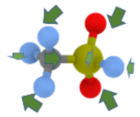
4	272.26	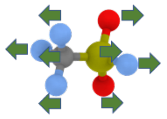	13	749.84	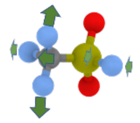
5	296.59	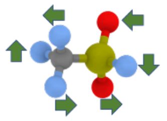	14	1064.31	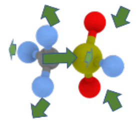
6	302.18	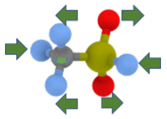	15	1168.00	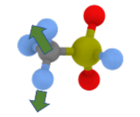
7	407.68	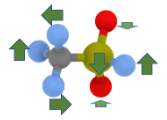	16	1176.60	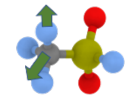
8	436.70	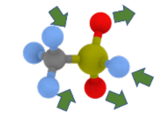	17	1186.37	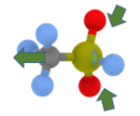
9	530.43	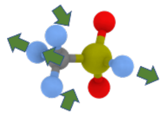	18	1397.05	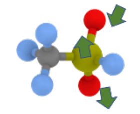

Table S4. Reaction pathways of CF_3SO_2F in the CF_3SO_2F/CO_2 and CF_3SO_2F/N_2 mixed gas.

No	CF_3SO_2F/CO_2	CF_3SO_2F/N_2
1	$CF_3SO_2F \rightarrow CF_4 + SO_2$	$CF_3SO_2F \rightarrow CF_4 + SO_2$
2	$CF_3SO_2F \rightarrow CF_3 + SO_2F$	$CF_3SO_2F \rightarrow COF_2 + SOF_2$
3	$CF_4 \rightarrow CF_3 + F$	$CF_3SO_2F \rightarrow CF_3 + SO_2F$
4	$CF_3 \rightarrow CF_2 + F$	$COF_2 \rightarrow COF + F$
5	$CF_2 \rightarrow CF + F$	$SOF_2 \rightarrow SOF + F$
6	$CF \rightarrow C + F$	$CF_4 \rightarrow CF_3 + F$
7	$SO_2 \rightarrow SO + O$	$CF_3 \rightarrow CF_2 + F$
8	$SO \rightarrow S + O$	$CF_2 \rightarrow CF + F$
9	$SO_2F \rightarrow SO_2 + F$	$CF \rightarrow C + F$
10	$SO_2F \rightarrow SOF + O$	$SO_2 \rightarrow SO + O$
11	$SOF \rightarrow SO + F$	$SO \rightarrow S + O$
12		$SO_2F \rightarrow SO_2 + F$
13		$SO_2F \rightarrow SOF + O$
14		$SOF \rightarrow SO + F$

Supplementary References

1. Zhang, Y. *et al.* DP-GEN: a concurrent learning platform for the generation of reliable deep learning based potential energy models. *Comput. Phys. Commun.* **253**, 107206 (2020).

2. Thompson, A. P. *et al.* LAMMPS - a flexible simulation tool for particle-based materials modeling at the atomic, meso, and continuum scales. *Comput. Phys. Commun.* **271**, 108171 (2022).

3. Tsyshevsky, R. V., Sharia, O. & Kuklja, M. M. Thermal decomposition mechanisms of nitroesters: ab initio modeling of pentaerythritol tetranitrate. *J. Phys. Chem. C* **117**, 18144–18153 (2013).

4. Kuklja, M. M., Tsyshevsky, R. V. & Sharia, O. Effect of polar surfaces on decomposition of molecular materials. *J. Am. Chem. Soc.* **136**, 13289–13302 (2014).

5. Wang, H., Zhang, L., Han, J. & E, W. Deepmd-kit: a deep learning package for many-body potential energy representation and molecular dynamics. *Comput. Phys. Commun.* **228**, 178–184 (2018).

6. Stukowski, A. Visualization and analysis of atomistic simulation data with OVITO–the open visualization tool. *Modelling Simul. Mater. Sci. Eng.* **18**, 015012 (2009).
