# Supplementary material for: Probing the thermal decomposition mechanism of CF3SO2F by deep learning molecular dynamics
Source: Commun Chem. 2025 Dec 19;9:40. doi: 10.1038/s42004-025-01847-x (PMC12824197; doi:10.1038/s42004-025-01847-x)
Supplement: Supplementary file 3 — Description of Additional Supplementary Files [file 42004_2025_1847_MOESM3_ESM.pdf]

Description of Additional Supplementary Files:

File: Supplementary Data 1

Description: source data underlying the graphs and charts in the main figures
